# Autozygosity and Genetic Differentiation of Landrace and Large White Pigs as Revealed by the Genetic Analyses of Crossbreds

**DOI:** 10.3389/fgene.2019.00739

**Published:** 2019-09-05

**Authors:** Luis Gomez-Raya, Wendy Mercedes Rauw, Jenelle R. Dunkelberger, Jack C. M. Dekkers

**Affiliations:** ^1^Department of Animal Science, Iowa State University, Ames, IA, United States; ^2^Departamento de Mejora Genética Animal, Instituto Nacional de Investigación y Tecnología Agraria y Alimentaria (INIA), Madrid, Spain

**Keywords:** autozygosity, runs of homozygosity, genetic differentiation, swine, crossbreeding

## Abstract

Genomic information from crossbreds is routinely generated for genomic evaluations. The objective of this study is to investigate autozygosity and genetic differentiation in Landrace by Large-White breeds by using the genotypic information of SNP arrays in 1,173 crossbreds. A maximum likelihood approach was developed to estimate the probability of autozygosity (*F_L_*). Regions of differentiation between breeds were investigated using *F_ST_* and the difference in allele frequencies between the two parental breeds (릌Δ) at each single-nucleotide polymorphism (SNP) position. A maximum likelihood approach was proposed to estimate allele frequencies in the parental populations. The average length of runs of homozygosity (ROH) across the genome was 3.91, 2.3, and 0.7 Mb for segments with at least 25, 15, and 5 SNPs, respectively. Average age to coalesce was 46, 414, and 388 years for segments with at least 25, 15, and 5 SNPs, respectively. The probability of autozygosity was not uniform along the crossbred genome, being higher at the center for most chromosomes. The correlation between autozygosity and distance to the closest telomere was positive and significant in most chromosomes, which could be attributed to the higher recombination rate near telomeres. We also report a relatively high negative correlation between probability of recombination (from a published map) and probability of autozygosity. It supports that structural characteristics of the chromosomes related to recombination rate determine autozygosity at each chromosomal position of the pig genome. The average is Δ across the genome was 0.17 (SD = 0.16). After testing for differences in allele frequencies between the parental breeds, there were 4,184 SNPs with a likelihood ratio test, LRT ≥ 32.02. The average *F_ST_* across the genome was 0.038 (SD = 0.059). There were 2,949 SNPs with *F_ST_* > 0.125. The correlation between estimates of *F_L_* and estimates of *F_ST_* across the genome was -0.10 (SE = 0.006). Analysis of the gene content of the genomic regions with the 2000 SNPs with highest LRT for *F_L_* and high *F_ST_* showed overrepresentation of genes with a regulatory function. Genes with biological functions associated with production, such as tissue development, anatomical structure, and animal organ development, were also overrepresented in regions with a high *F_ST_*.

## Introduction

Domestication of the pig took place some 9,000 years ago and occurred multiple times (Giuffra et al., 2000). After domestication, a large variety of local types were created, both in Europe and in China. These local types were adapted to the environmental conditions where they were raised and were independently selected for behavior and conformation traits, until the creation of modern breeds in the 19th century (Amills et al., 2010). The Large White breed was recognized as a genuine breed in England in 1868, and its first herd book was published in 1884 (Jones, 1998). The Landrace breed originated in Denmark and was then exported to England. The creation of breeds required breeding in closed populations, in which both selection and genetic drift altered the genetic makeup of those populations by modifying allele frequencies at loci throughout the genome. The creation of breeds led to their subsequent use in crossbreeding, which has been widely used to exploit heterosis in both animals and plants. Molecular techniques can now help to identify similarities and differences in today´s populations or breeds. Evolution of differences in the genetic makeup of breeds can be attributed to: 1) changes in allele frequency caused by genetic drift in different directions (as estimated by the *F_ST_* of Wright), 2) changes in allele frequencies by (natural or artificial) selection in one or both breeds, and 3) a combination of these two processes. Nevertheless, alleles are not necessarily segregating at a different frequency in the two breeds at all loci. For example, directional or stabilizing selection may be acting with the same intensity at the same allele (or chromosomal fragment) in the two breeds over time.

The presence of autozygosity in crossbreds can be revealed by runs of homozygosity (ROH), which are defined as continuous and uninterrupted stretches of homozygous DNA sequences in diploid state (Gibson et al., 2006). Varying lengths of ROH provide generational information about inbreeding levels in a reference population (Curik et al., 2014). Long ROH fragments imply also recent autozygosity (Keller et al., 2011; Gomez-Raya et al., 2015). ROH (measured in genetic mapping units) can be used to trace back the age where the two chromosome segments coalesce in an ancestor (Thompson, 2013). ROH are not uniformly distributed across the genome but are more numerous in some regions, termed ROH islands (Nothnagel et al., 2010) or ROH hotspots (Pemberton et al., 2012). ROH islands are often in chromosomal regions with reduced effective population size harboring genes under purifying selection but also in regions with low recombination rates (Charlesworth et al., 1993; Gibson et al., 2006; Pemberton et al., 2012; Kardos et al., 2017). For example, chromosomes 3, 4, and 14 contain an abundance of ROH in European human populations (Nothnagel et al., 2010). In contrast, ROH deserts or colds spots are regions where ROH are rare and could represent regions of increased diversity (Pemberton et al., 2012). In farm animals, the presence of ROH islands and deserts could be more pronounced due to current breeding practices, including selection and the small effective size of breeding populations. In several cattle breeds, ROH hotspots have also been identified (Gurgul et al., 2016a; Gurgul et al., 2016b; Szmatoła et al., 2016). Most work investigating selection signatures using ROH has made use of outbred populations in which ROH result from inbreeding and selection (Kim et al., 2013). An alternative is the use of crossbreds, which are routinely used for commercial production and may provide information on the genomic differences (or lack of thereof) in the parental breeds. ROH in crossbreds have a different meaning than in outbred populations since persistence of autozygosity could be understood as selection for the same gene (or chromosomal region) in the two parental breeds. Homozygosity due to inbreeding is less likely because it would require that drift fixed the same allele (or chromosomal region) in the parental breeds. Nevertheless, little work on ROH has been done using crossbred animals, with the exception of Howard et al. (2016), who observed that long stretches (5 to 10 Mb) of ROH that are present in the parents persist in the crossbred offspring. Thus, genomic analyses of crossbreds may shed light on the evolution and dynamics of chromosomal regions and persistence of the same long ROH in the parental populations.

The objective of this study is to investigate autozygosity and genetic differentiation in two parental breeds (Large White and Landrace) using the genotypic information from their crossbred progeny. The specific objectives were: 1) determining levels and regions of autozygosity and its relationship with recombination rate in crossbred progeny, 2) determining differences in allele frequency across the genome between the two parental breeds, and 3) determining genetic differentiation in Large White and Landrace by investigating the genomic distribution of Wright’s *F_ST_* across the genome in the two breeds.

A method to estimate allele frequencies in the parental lines based on crossbred genotypes was developed because the genotypic information of the purebred parents was not available.

## Materials and Methods

### Animal Material and DNA Genotyping

F_1_ Landrace x Large White crossbred barrows from two companies with 605 (company A) and 568 (company B) pigs, respectively, were used. Data were from six Porcine Reproductive and Respiratory Syndrome (PRRS) virus challenge trials with about 200 piglets each: PHGC17 (from 15 boars and 49 sows), PHGC18 (from 12 boars and 51 sows), PHGC21 (from 10 boars and 81 sows), PHGC23 (from 11 boars and 52 sows), PHGC24 (from 10 boars and 46 sows), and PHGC25 (from 9 boars and 72 sows). It was assumed that the parental populations from these companies are representative of the Landrace and Large White breeds. DNA was isolated from blood using a standard phenol/chloroform protocol and genotyped with either the Illumina Porcine SNP80 BeadChip or the SNP50 BeadChip and the Infinium HD Assay Ultra protocol (Illumina Inc.). All common SNPs of the two arrays were mapped to the Sscrofa11.1 assembly. Genotypes were called with GenomeStudio software (Illumina). Quality control of genotypes was performed using the following criteria: average call rate for a SNP >0.70; individuals with SNPs that had a genotype call rate <0.70 were assumed not genotyped for those SNPs; SNPs located on sex chromosomes and those not mapped in Sscrofa11.1 were also removed. After editing, the data consisted of genotypes for 32,659 SNPs on 1,173 crossbred pigs. There were 764 SNPs with frequency less than 0.05. In general, SNPs were evenly distributed across the swine genome. A program in Fortran 90 was written to identify ROH in the genome for each individual. The program searched contiguous and homozygous SNPs within each of the 18 autosomal chromosomes. For computing age to coalesce a minimum number of 5, 15, or 25 SNPs were required for declaring a stretch of DNA as a ROH. A fragment was not considered a ROH if there was one or more heterozygous SNPs in the stretch of DNA. The number of generations back to the common ancestor, *g*, of ROH fragments was estimated by solving for g the equation *L* = 100/2g, where *L* is the ROH length in *cM* (Thompson, 2013). Physical distances in *Mb* were converted to *cM* assuming 1 *cM* = 1 *Mb*. The length in *cM* for each segment was obtained by multiplying by 100 the recombination fraction obtained after using the inverse of the Kosambi map function: $\theta =\frac{1}{2}(\frac{e^{d}-1}{e^{d}+1})$, where d is the length of each ROH in *Mb*. The age to coalesce for each ROH segment was obtained by assuming a generational interval of 2 years. For any other analysis, ROH segments were defined by 25 or more contiguous and homozygous SNPs.

### Maximum Likelihood Estimation of Autozygosity Based on ROH

ROH can be used as a measure of autozygosity in an individual. If autozygosity occurs randomly, then any SNP position within a chromosome should have the same chance of being autozygous. A statistic to estimate the local probability of autozygosity in a population based on N individuals is *F_L_* (Kim et al., 2013):

$$F_{L}=\frac{\sum_{i=1}^{N}h_{i}}{N}$$

where *h_i_* is the ROH state of the *i-th* individual (*h_i_* =1 if the *i-th* individual contains a ROH at that position and *h_i_* = 0, otherwise), which was used by Kim et al. to compare cattle breeds. An alternative proposed in the present article is to compare *F_L_* with its expected distribution under the hypothesis that ROH status *h_i_* =1 or being autozygous is equally likely for all SNP positions within a chromosome. The likelihood of the number of animals with ROH status *h_i_* =1 at a given position under the null hypothesis is assumed to follow a binomial density with likelihood:

[1] $$L\left(\hat{F_{L}}\text{|}N,m\right)=\frac{N!}{m!(N-m)!}{\left(\hat{F_{L}}\right)}^{m}{\left(1-\hat{F_{L}}\right)}^{N-m}$$

where *m* is the number of animals with status *h_i_* =1 at a given SNP position. The maximum likelihood of $\hat{F_{L}}$ is *m/N* and the sampling variance of $\hat{F_{L}}$ is $V\left(\hat{F_{L}}\right)=\;\frac{\left(\hat{F_{L}}\right)\left(1-\left(\hat{F_{L}}\right)\right)}{N}$. Under the null hypothesis, the probability for a SNP to be autozygous in the *i-th* individual is equal to the chromosomal inbreeding coefficient for that individual, $F_{i}^{\text{ROH}}$, which is calculated by summing the number of SNPs over all ROH fragments and dividing it by the total number of SNPs genotyped in that chromosome. To reduce variation, the individual $F_{i}^{\text{ROH}}\;$ was pooled across individuals by $F_{0}^{ROH}=\frac{\sum_{i=1}^{N}F_{i}^{ROH}}{N\;n_{snp}}$, with *n*_snp_ being the number of SNPs genotyped on that chromosome. Under the alternative hypothesis, $\hat{F_{L}}$ is either higher (islands or hot spots) or lower (deserts or cold spots) than under the null hypothesis of equal probability of autozygosity at any SNP position for each chromosome. Departures from the null hypothesis were tested by a Likelihood Ratio Test (LRT):

[2] $$\begin{array}{l} LRT=2[m\;\;ln\left(\hat{F_{L}}\right)+(N-m)\text{ln}\left(1-\hat{F_{L}}\right)\\ -(m\;lnF_{0})-(N-m)ln(1-F_{0}^{ROH})] \end{array}$$

A LOD score was constructed as:

$$LOD=log_{10}\frac{L\left(\hat{F_{L}}\right)}{L\left(F_{0}^{ROH}\right)}$$

Under the null hypothesis, LRT is distributed as a χ^2^ with one degree of freedom. In our analysis, a test was declared significant after using a Bonferroni correction to account for multiple testing with a genome-wide significance level of 0.001 (the threshold of the χ^2^ was 32.02). In addition, and to estimate sample size needed to detect departure from the null hypothesis, statistical power was computed with the package pwr in R (https://www.r-project.org/).

### Estimation of Genetic Differentiation in the Parental Lines Using Genotypic Information From Crossbreds

The estimation of genetic differentiation requires the availability of estimates of allele frequencies in the parental populations. Methods for the estimation of allele frequencies within a population are the direct method or the maximum likelihood for unrelated individuals (Adrianto and Montgomery, 2012), and the maximum likelihood when there is a family structure (Boehnke, 1991). There is methodology for inferring admixture proportions from molecular data, which may include the estimation of allele frequencies in the putative parental populations (Falush et al., 2003). These methods require estimation of many unknown parameters and are not the most appropriate for the estimation of allele frequencies in crossbreds. Estimation of allele frequencies using crossbred data can be carried out by maximum likelihood (Bovenhuis and Van Arendonk, 1991). In line with their work, we propose a maximum likelihood method to estimate allele frequencies in the parental lines, and its sampling variance using genotypic information from the crossbreds (**Appendix**). The method is based on putative departures from Hardy-Weinberg equilibrium conditions in the crossbreds. A computer simulation was carried out to validate the proposed method to estimate allele frequencies. Genotypes from 1,173 crossbred animals were generated by draws of the uniform distribution assigning alleles A or B with a probability corresponding to the simulated allele frequencies in each parental breed. For each replicate, estimates of allele frequency in the two populations and corresponding hypothesis testing by a Likelihood Ratio Test (*LRT*) were performed (see Appendix). In addition, the standard deviation (SD) of the estimates of the allele frequencies was obtained by both 1) using the standard deviation of the estimates of allele frequencies across the 10,000 simulated replicates and 2) taking the square of the estimates of variance accordingly to the formulae in the Appendix. Empirical power was computed using a significance level of 0.01, which corresponds to the 100*th* larger *LRT* result, obtained in the 10,000 replicates when simulating the null hypothesis (allele frequencies of 0.5 in the two breeds). In our simulation experiment, this value was 5.66. Then, power was computed as the proportion of replicates that were higher than 5.66 for each of the simulation experiments: (1) *f_A_* = 0.1, *f_B_* = 0.9; (2) *f_A_* = 0.3, *f_B_* = 0.7; (3) *f_A_* = 0.35, *f_B_* = 0.65; and (4) *f_A_* = 0.4, *f_B_* = 0.6. The grid search method was used to solve the likelihood equations. In this method, the likelihood is evaluated at short intervals of the two allele frequencies throughout the entire range of possible values (0 to 1). As shown in the Appendix, there are two solutions to likelihood equations, which makes it difficult to assign each estimate of allele frequency to each parental population. To overcome this problem, the grid search method was performed starting the A allele frequency as in 0 and ending in 1. Then, grid search of the B allele frequency from 0 to 1 was performed within each of the A allele frequencies. The estimate of allele frequencies was the first maximum found by the grid search method. It allowed assigning allele frequencies to the two parental breeds when the difference in allele frequency is large, since the grid search method visits the same allele frequencies (A and B) in the same order in each replicate. For the null hypothesis, the estimates of the allele frequencies were pooled since both were 0.5.

Genetic differentiation between the two parental breeds, A and B, was investigated by:

a) $∆=abs(\hat{\;f_{A}}-\hat{\;f_{B})}$ at each SNP, where $\hat{\;f_{A}}$ and $\hat{\;f_{B}}$ are the estimates of allele frequencies in the two breeds as obtained with the method described in the **Appendix**. The sampling variance of the estimates of allele frequencies in the parental populations is also derived in the Appendix. Δ is a measure of how genetically distant the parental breeds are at a particular SNP location. Testing of the hypothesis was carried out using a likelihood ratio test as described in the Appendix. A difference in allele frequencies between parental breeds, together with dominant gene action, is the most accepted theory for heterosis (Davenport, 1908), which has been supported with experimental data (Xiao et al., 1995).

b) The *F_ST_* method of Wright (Wright, 1943) for each SNP using the estimates of allele frequencies in the parental lines, as described in the Appendix. The *F_ST_* was calculated at each SNP as $F_{ST}=\frac{\sigma_{f}^{2}}{f\left(1-f\right)}$, where *f* is the average of $\hat{\;f_{A}}$ and $\hat{\;f_{B}}$ at that SNP, and $\sigma_{f}^{2}$ is the variance in the frequency of the allele among the parental breeds A and B.

### Relationship Between *F_L_*, *F_ST_*, and Δ With Distal Chromosomal Distance

High values of *F_L_* were generally observed in the central parts of each chromosome after genome-wide estimation of this statistic. There are several reasons why autozygosity may be higher at the center of a chromosome, including the region being near the centromere, where recombination is repressed. Because it was difficult to determine the exact map location of the centromere, the distance to the closest distal part of the chromosome was used for each SNP in each chromosome to investigate if the relationship between the distance to the closest telomere and the probability of autozygosity followed the same pattern across chromosomes. The relationship between *F_ST_* and Δ with distal chromosomal distance was also investigated. A linear regression model was fitted using *F_L_* (or *F_ST_* or Δ) at each SNP within chromosome as the dependent variable and the distance to the closest telomere as the independent variable. Correlations between *F_L_* (or *F_ST_* or Δ) and the distance to the closest telomere per chromosome and genome-wide are reported.

### Correlation Between Local Recombination Rate and *F_ST_* and *F_ST_* Across the Swine Genome

The results of the analyses of this study suggested that variation in the recombination rate across the genome could be related to the local autozygosity. A literature review revealed that there is a published map of the recombination rate across the pig genome (Tortereau et al., 2012). In this map, the recombination rate is provided in intervals of approx. 1 Mb. The recombination rate map was built using four different crosses and version 10.2 of the assembly pig map (Tortereau et al., 2012). The following steps were carried out to estimate the correlation between the recombination rate and the probability of autozygosis: 1) merge files containing our estimates of autozygosity together with the map of Tortereau et al. (2012) using SNP name in version 10.2 of the genome assembly of *Sus Scrofa*; 2) sort the file according to chromosome, and position within chromosome; and (3) assign the recombination fraction of a given Mb from the map of Tortereau et al. (2012) to each SNP with estimates of autozygosity within the given interval. The same process was carried out with estimates of *F_ST_*. After this process, we were able to assign 29,315 SNPs with figures of recombination fraction to our estimates of *F_L_* and *F_ST_*. A linear regression between recombination rate and *F_L_* (or *F_ST_*) was carried out for each of the 18 autosomal chromosomes. A genome-wide linear regression between recombination rate and *F_L_* (or *F_ST_*) was also carried out. In addition to these analyses, a linear regression was performed between estimates of *F_L_* and *F_ST_*.

### Gene Content in Regions With High or Low Autozygosity and High *F_ST_*

The 2,000 SNPs with the highest *LRT*, were selected to further investigate the biological functions of the genes located within those regions. The number of 2,000 SNPs was decided based on a trade-off between SNPs associated to high autozygosity and power to detect association with biological functions. Due to varying allele or haplotype frequencies within the same string of consecutive SNPs, not all SNPs were significant in a given autozygosity chromosomal region. To declare a chromosomal region as high or low autozygosity the following rules were applied after ordering each set of 2,000 SNPs: 1) the physical distance between two consecutive and significant SNPs was less than 2 Mb, 2) the value of *F_L_* for each of two consecutive SNPs was either higher (region of high autozygosity) or lower (region of low autozygosity) than expected for that chromosome under the null hypothesis $F_{0}^{ROH}$, and 3) there were at least 10 SNPs in the region. Version 11.1 of Sus Scrofa of biomart (http://www.ensembl.org/biomart/martview/) was used to identify putative genes located in regions of high or low autozygosity and high genetic differentiation. A test of overrepresentation for the GO complete biological processes was carried out using the PANTHER software (http://pantherdb.org/) to investigate the biological function of genes in high or low regions of autozygosity. The same approach was used for the 2,000 SNPs with the highest *F_ST_*. The over-representation test applies a Bonferroni correction to account for multiple testing. Only significant biological processes with P values < 0.001 are presented for high and low autozygosity and only those with P values < 0.0001 for regions with high *F_ST_*.

## Results

### Autozygosity in Landrace and Large White Crossbreds

**Figure 1:**
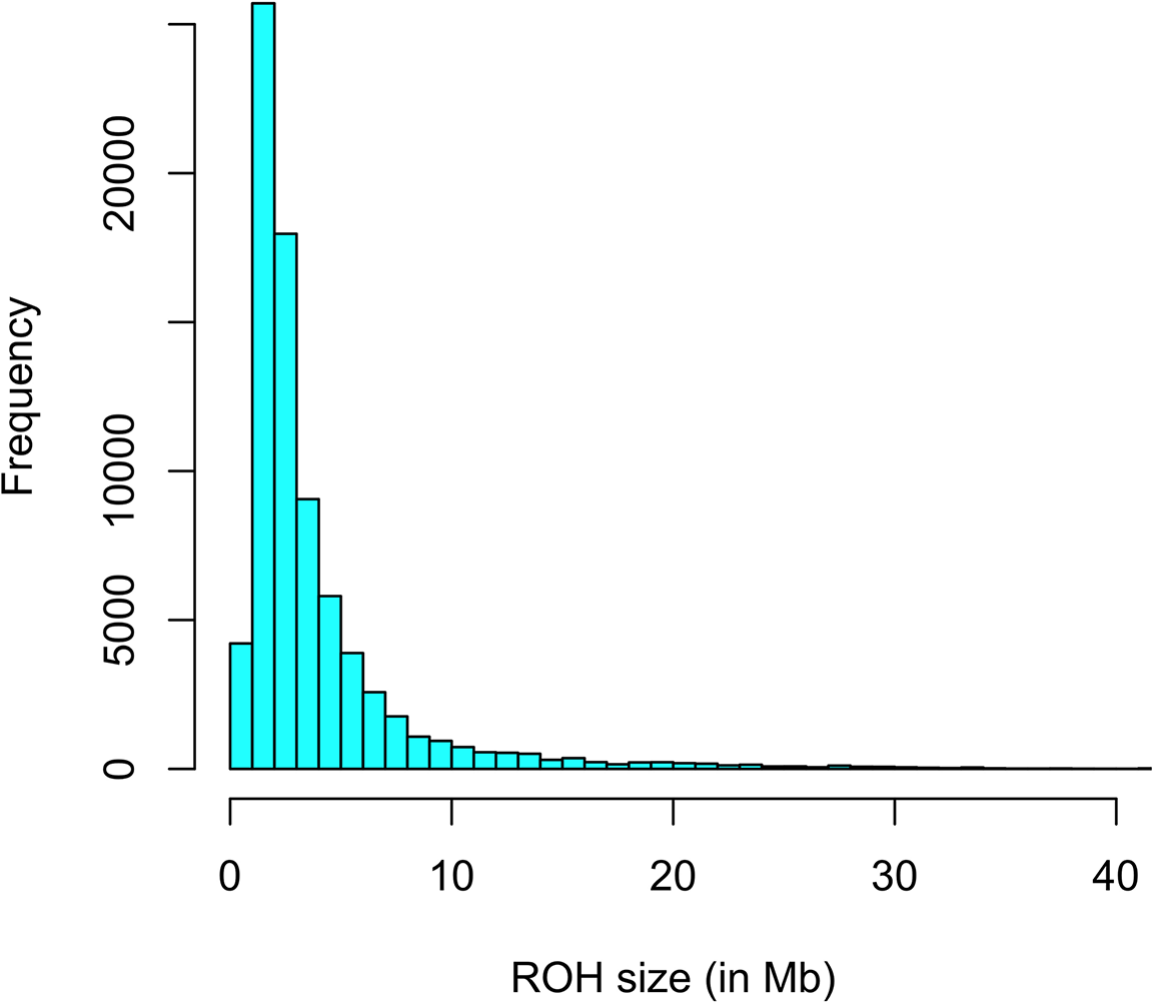
Distribution of the Size of ROH among 1,173 Landrace x Large-White crossbreds.

**Figure 2:**
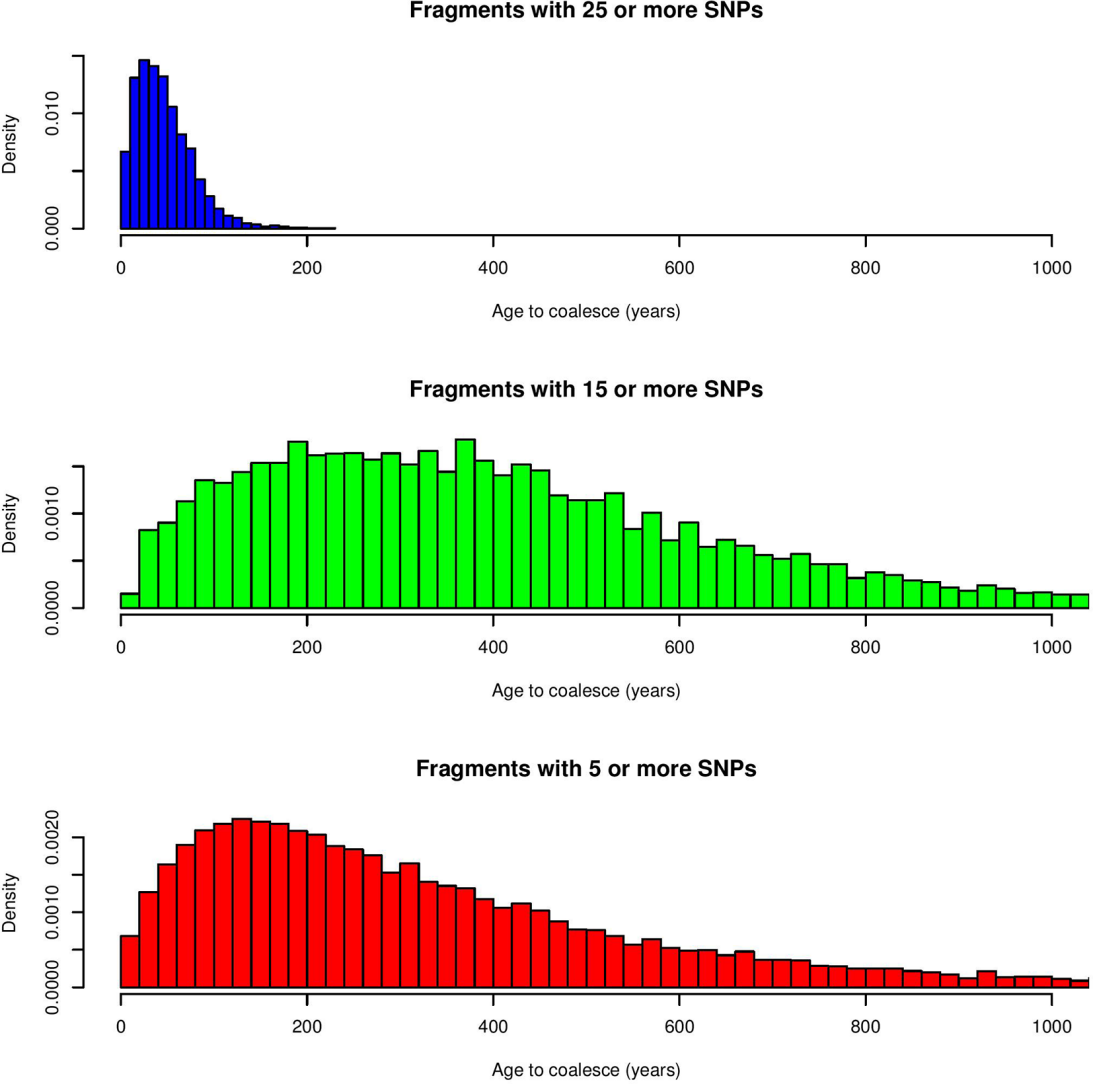
Distribution of the age of ROH fragments to coalesce to a common ancestor (in years) for a minimum of 25, 15 or 5 SNPs.

**Figure 3:**
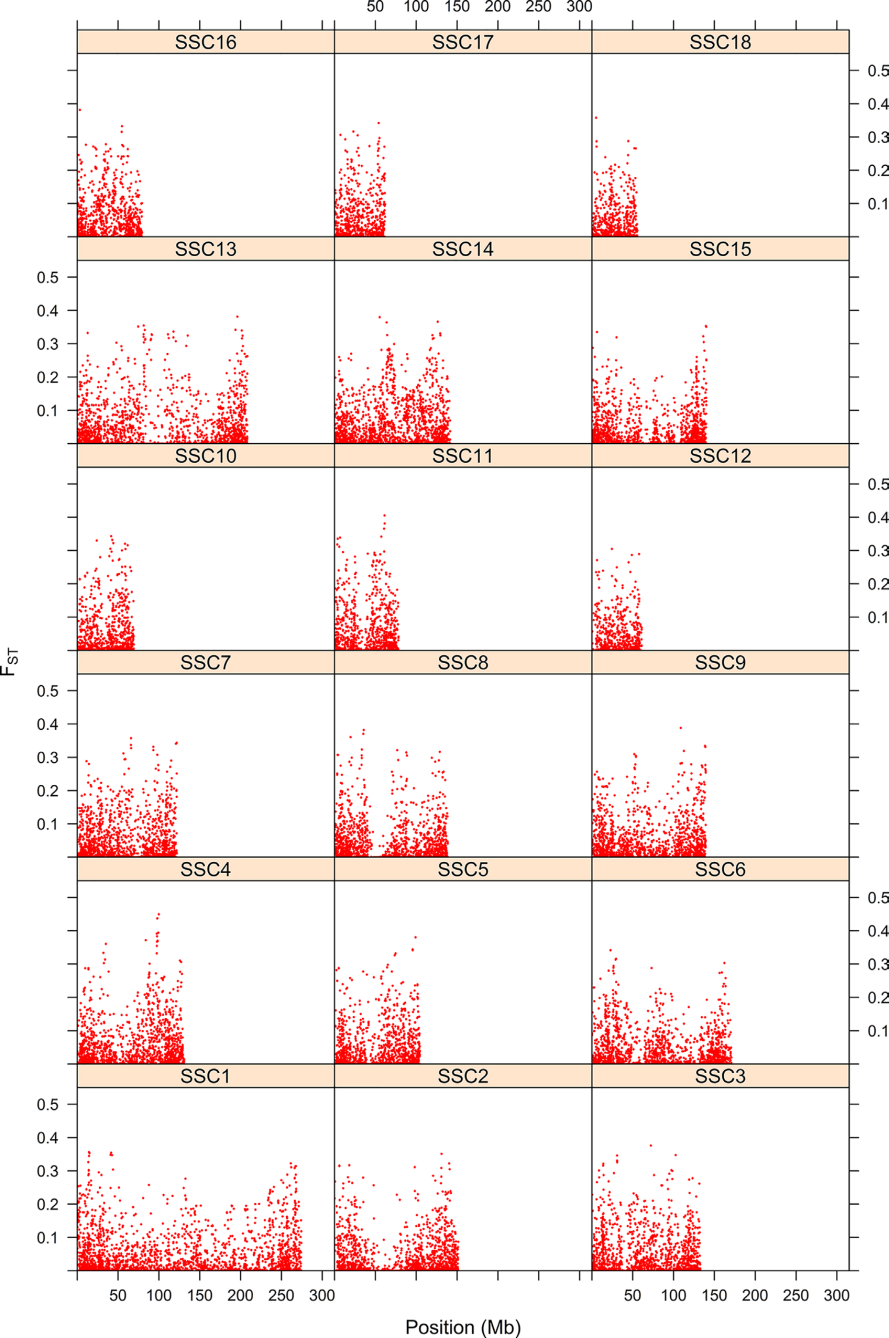
Map of the probability of autozygosity (*F_L_*) in Landrace x Large-White crossbreds. The y-axis represents the probability of autozygosis for each of the 18 swine chromosomes. The x-axis indicates the SNP position.

**Figure 4:**
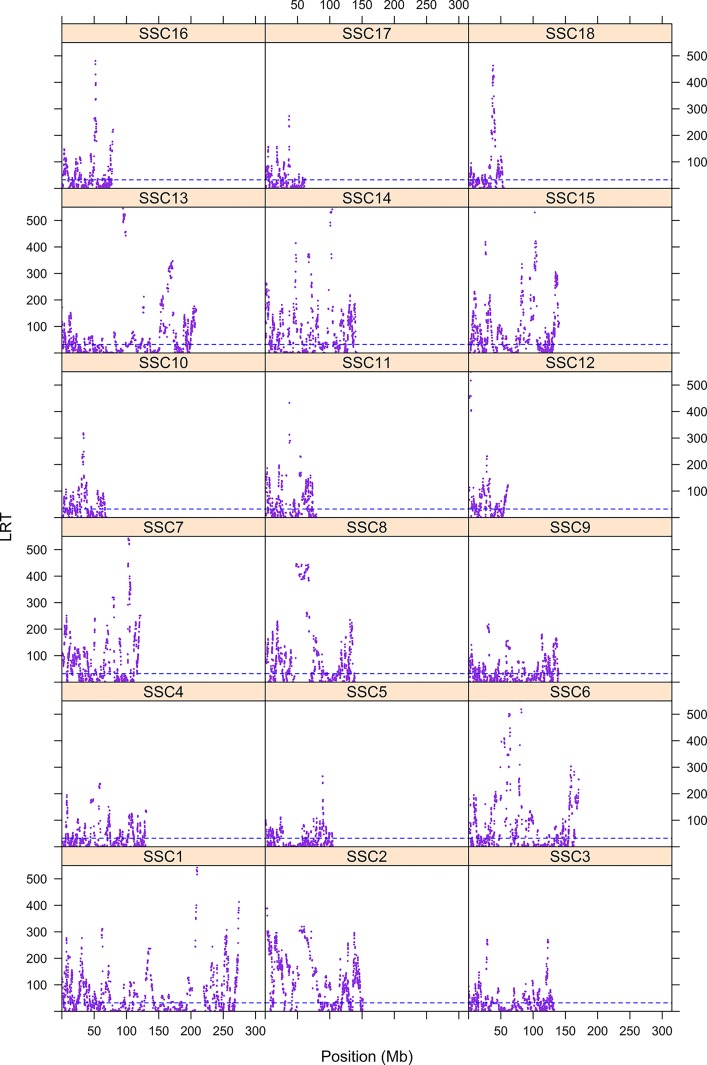
Manhattan plot of autozygosity of Landrace x Large White crossbreds. The y-axis represents the LRT for each for the 18 swine chromosomes. The x-axis indicates the SNP position.

The first step consisted of computing statistical power to evaluate if sample size was sufficient to detect autozygosity. Results showed that a sample size of 1,114 (two-side test) is required to detect a difference of *F_L_* = 0.16 (alternative hypothesis of high autozygosity) from *F_L_* = 0.12 (null hypothesis) with a statistical power of 0.90 at a significance level of 0.01. Consequently, the sample size of our data was sufficient to detect deviations from the expected autozygosity under the null hypothesis. There were 32,659 SNPs genotyped for all individuals and the data analyses revealed a distribution of the size of ROH fragments (with 25 or more SNPs) as shown in **Figure 1**. The average length of ROHs was 3.91, 2.35, and 0.71 Mb when assuming 25, 15, and 5 (or more SNPs) in each ROH, respectively. The largest ROH segment was 129.4 Mb, which may indicate some recent non-intended consanguineous mating. **Figure 2** shows the distribution of the age in which ROH segments coalesce to a common ancestor. The average number of generations back to the common ancestor is 46, 415, and 388 years when requiring a minimum of 25, 15, or 5 SNPs, respectively. There is a large impact on the assumed length of the ROH on the estimates of the age to coalesce. A map of autozygosity and corresponding Manhattan plots (*LRT* along chromosomal position) are depicted in **Figures 3** and **4**, respectively. Lod score maps are also provided in **Supplemental Figure 1** because lod scores are widely used in genetic studies. For most chromosomes, the probability of autozygosity had a clear trend to be larger in the central parts of the chromosome, whereas the opposite trend was observed for the distal parts of most chromosomes, which is consistent with a higher recombination rate near telomeres. Two estimates of autozygosity were also obtained after analyzing independently the pigs of the two companies. The two companies’ stocks are mostly isolated genetically. The correlation between the estimates of autozygosity, *F_L_*, obtained in the data set of each of the two companies, was 0.50 (SE 0.005). This supports the hypothesis that the probability of autozygosity depends on the chromosome position and may be influenced by chromosomal physical characteristics or by the local recombination rate.

### Differentiation Between Landrace and Large White

**Figure 5:**
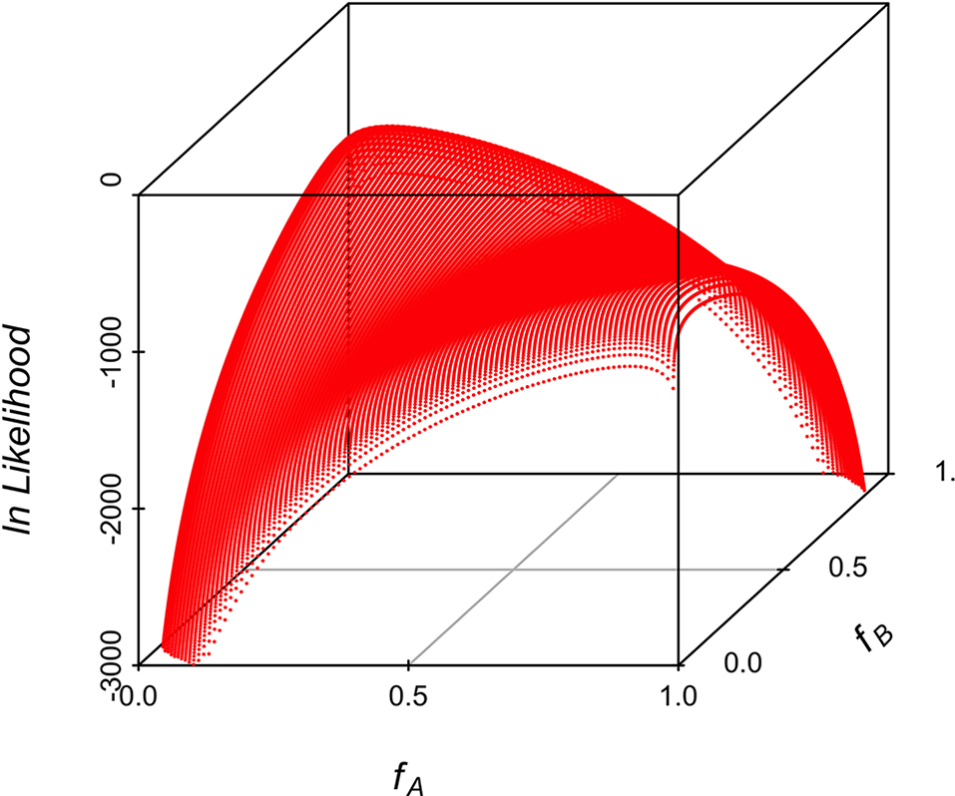
3D scatterplot of the natural logarithm of the likelihood along the allele frequencies in the two parental populations for one example illustrating the two maxima corresponding to the allele frequencies of the two parental populations. The simulated allele frequency was 0.1 and 0.9 in the two parental breeds.

Maximum likelihood estimates of allele frequencies in the parental populations (required to estimate differentiation) always yielded two solutions, corresponding to the two alternatives for assigning frequencies to the parental breeds. **Figure 5** shows the likelihood for an example of one replicate simulating allele frequencies of 0.1 and 0.9 in the parental breeds. The results in **Figure 5** show that the associated likelihood function has two maxima, corresponding to the two possibilities of assigning the estimates of allele frequencies to the two parental populations, i.e., it is not possible to resolve which frequency corresponds to which parental population. However, identification of the population and its corresponding frequency is not necessary because the equations for genetic differentiation are either based on the difference or on the product of the allele frequencies in the two populations. The results of the simulation for the 10,000 replicates had always two maxima and are depicted in **Table 1**. The average of the estimates of allele frequencies were very similar to the simulated allele frequencies when the difference between allele frequencies in the parental breeds was large. Assigning estimates of allele frequency to each breed was not always correct when the difference in allele frequency between the parental breeds is small. The observed standard deviation of estimates of allele frequencies was very similar to the predicted values using the formulae in the Appendix as long as the difference in allele frequency in the parental breeds was not large. Empirical statistical power shows that the sample size of this study is enough to detect significant associations for a difference in allele frequency of 0.2.

**Table 1:** Average estimates over replicates for allele frequencies and LRT, observed and predicted standard deviation for allele frequencies (SD), and statistical power at significance level of 0.01 of the computer simulation for varying allele frequencies in the two populations at crossing.

Simulated frequencies	Average over replicates			Observed SD for *f_A_*	Predicted SD for *f_A_*	Power
	*f_A_*	*f_B_*	LRT			
*f_A_* = 0.1 *f_B_* = 0.9	0.100	0.900	521.44	0.0093	0.0093	1.000
*f_A_* = 0.3 *f_B_* = 0.7	0.301	0.699	31.07	0.0204	0.0203	0.999
*f_A_* = 0.35 *f_B_* = 0.65	0.352	0.648	10.48	0.0286	0.0262	0.752
*f_A_* = 0.4 *f_B_* = 0.6	0.408	0.592	2.83	0.0432	0.0378	0.150
*f_A_* = 0.5 *f_B_* = 0.5	0.500		0.51			

**Figure 6:**
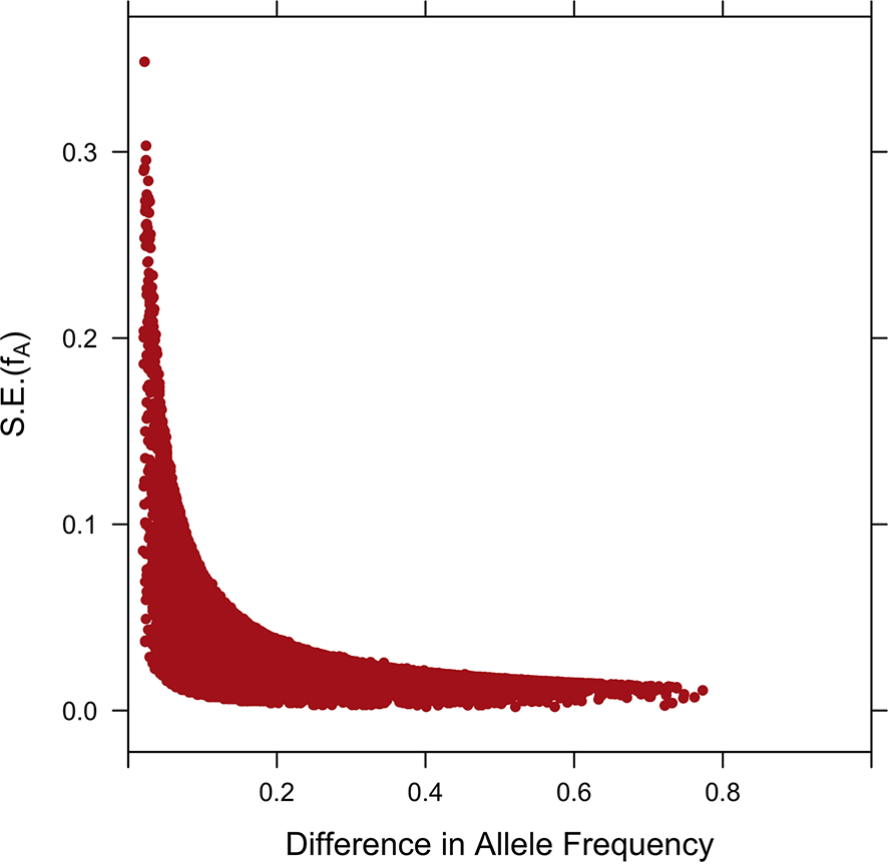
Plot of the standard errors S.E.(*f_A_*) against the difference of estimates of SNP allele frequencies in parental breeds.

Standard errors of the estimates of allele frequencies estimated using the 1,173 crossbreds are depicted in **Figure 6**. The sampling variance was large when allele frequencies were similar in the two parental populations, which is consistent with the nearly flat likelihood function in those situations. For a difference in allele frequencies between the two populations of 0.10, the standard error ranged from 0.00 to 0.07.

**Figure 7:**
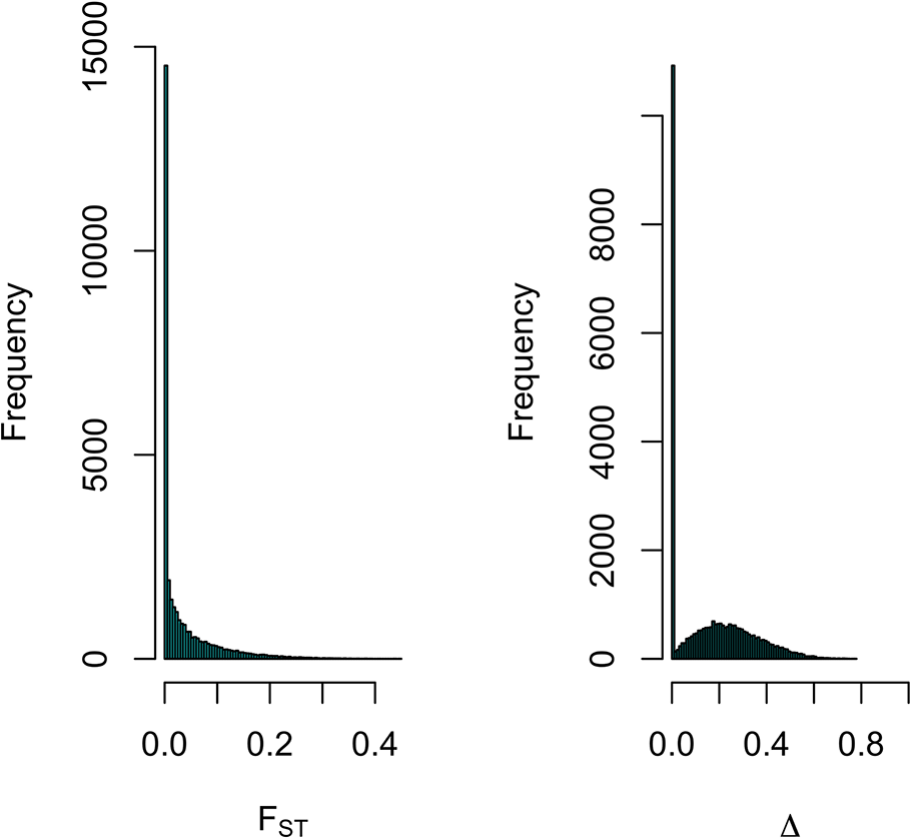
Distribution of *F_ST_* and Δ (difference in allele frequency) for SNPs across the genome for estimating differentiation between Large-White and Landrace populations.

**Figure 8:**
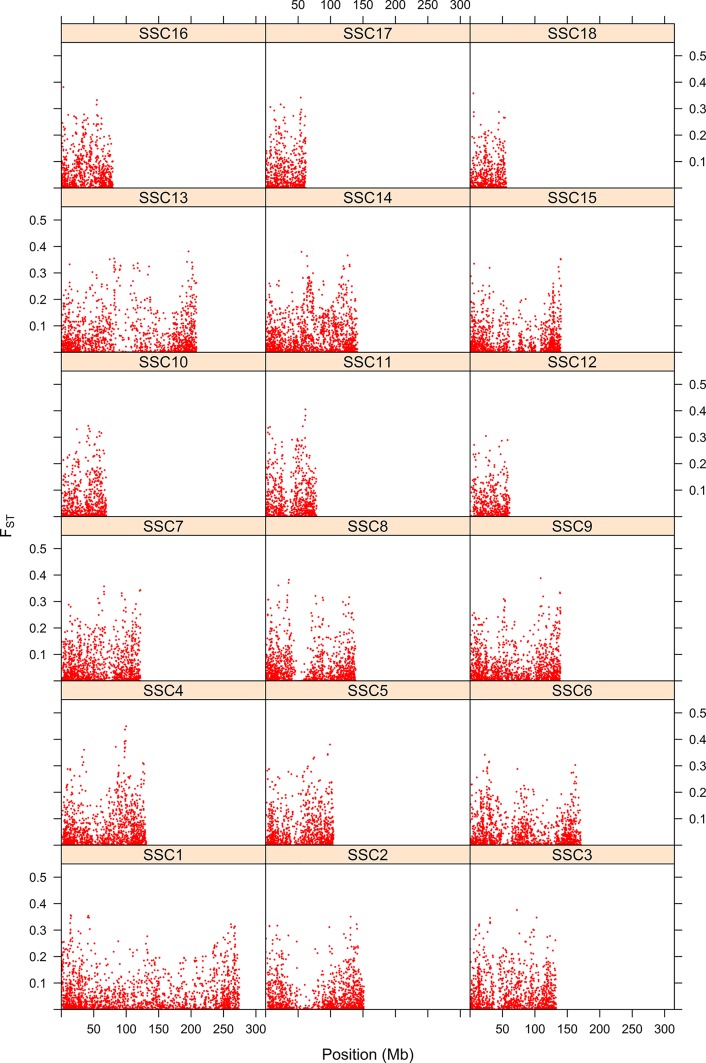
Map of differentiation of Landrace x Large-White using the FST statistic. The y-axis represents the FST statistic along each position for the 18 swine chromosomes. The x-axis indicates the SNP position.

Differentiation between parental populations was investigated using *F_ST_* and Δ (absolute value of the difference in allele frequencies), with estimates of allele frequencies in the parental populations. The mode of the distributions of *F_ST_* and Δ showed that allele frequencies were not different in the two parental breeds for a large number of SNPs (**Figure 7**). Thus, there were 16,464 SNPs with *F_ST_* < 0.01 and when testing for Δ, there were 15,451 SNPs with a LRT < 1. **Figure 8** shows the genome-wide distribution of *F_ST_*. The average *F_ST_* across the genome was 0.038 (SD = 0.059). There were 2,949 SNPs with an *F_ST_* > 0.125, which illustrates regions of differentiation between the two breeds. **Supplemental Figure 2** shows a Manhattan plot for Δ. The average Δ across the genome was 0.17 (SD = 0.16). There were 4,184 SNPs with a LRT > 32.02 (significant at P < 0.001 after Bonferroni correction for multiple testing). There were some highly significant chromosomal regions for differentiation (e.g., SSC4, **Supplemental Figure 2**). The correlation between *F_ST_* and Δ was 0.73 (SE ∼ 0.004), suggesting similarities between both estimates of differentiation between the Landrace and Large White breeds.

### Relationship Between *F_L_*, *F_ST_*, and Δ With Distal Chromosomal Distance

The correlation of probability of autozygosity (*F_L_*) with distance to the closest telomere was positive and highly significant for all chromosomes (**Table 2**), except for chromosomes 3, 12, and 17. The pattern of correlations in **Table 2** illustrates that the relationship between autozygosity and distance to the closest telomere differed by chromosome, which suggests that each chromosome may have distinct physical attributes that facilitate the conservation of haplotype regions in the parental breeds, leading to regions of autozygosity in the crossbreds. The correlation of *F_ST_* and Δ with distance of the SNP to the closest distal part of the chromosome differed greatly among chromosomes, being positive and significant for chromosomes 3, 14, and 16, and negative and significant for chromosomes 1, 2, and 15 (**Table 2**). These results suggest that selection and genetic drift may have acted differently in the different chromosomes.

**Table 2:** Pearson correlation and p-values of *F_L_*, *F_ST_*, and Δ at each SNP with its distance (in Mb) to the closest telomere by chromosome.

Chromosome	Number of SNPs	*F_L_*	*F_ST_*	Δ
1	3406	0.28****	–0.11****	–0.10****
2	2160	0.68****	–0.13****	–0.17****
3	1942	–0.10****	0.12****	0.18****
4	2078	0.39****	0.03	0.02
5	1587	0.16****	0.02	–0.02
6	2279	0.42****	–0.03	–0.03
7	1940	0.37****	0.04	0.00
8	1989	0.43****	–0.01	–0.02
9	2177	0.25****	–0.06**	–0.04
10	1063	0.55****	0.04	0.04
11	1265	0.29****	0.05	0.01
12	835	0.03	0.05	0.03
13	2565	0.31****	0.07***	0.02
14	2248	0.55****	0.24****	0.28****
15	1987	0.44****	–0.14****	–0.14****
16	1262	0.54****	0.23****	0.24****
17	989	0.02	–0.02	–0.01
18	887	0.26****	0.07	0.14****
Genome-wide	32,659	0.38****	–0.00	–0.02**

****P < 0.0001; ***P < 0.001; **P < 0.01.

Standard errors were between 0.02 and 0.03.

### Correlation Between Local Recombination Rate and *F_L_* and *F_ST_* Across the Swine Genome

**Table 3** shows the Pearson correlations between estimates of the recombination rate (Tortereau et al., 2012) and *F_L_* or *F_ST_*. The correlations between recombination rate and F_L_ were negative, highly significant and ranged from -0.16 to -0.52 in the 18 autosomal chromosomes. The genome-wide correlation was -0.366. These results suggest that autozygosity increases in chromosome regions with a low rate recombination and that those areas depend on structural physical attributes of chromosomes since the animals in the map of Tortereau et al. (2012) and in our study are different and unrelated. On the contrary, the correlation between recombination rate and estimates of *F_ST_* were positive (SSC1, SSC2, SSC6, SSC15), negative (SSC3, SSC14) or close to zero (rest of autosomal chromosomes). A genome-wide correlation between recombination rate and *F_ST_* was close to zero and not significant. The correlation between estimates of *F_L_* and *F_ST_* across the genome was -0.095 (SE = 0.006), which supports a significant negative relationship between these two measures. **Supplemental Figure 3** shows the distribution of *F_L_* and *F_ST_* per chromosome. The observed general trend is that SNPs with high *F_L_* have low *F_ST_* and vice versa. However, there are no SNPs with high values for both *F_L_* and *F_ST_*.

**Table 3:** Pearson correlation between recombination rate and *F_L_* and *F_ST_*.

Chromosome	*F_L_*	SE	*F_ST_*	SE
1	–0.383****	0.017	0.115****	0.018
2	–0.521****	0.019	0.140****	0.022
3	–0.164****	0.024	–0.083***	0.024
4	–0.425****	0.021	–0.029	0.023
5	–0.251****	0.026	0.027	0.026
6	–0.390****	0.020	0.078***	0.022
7	–0.406****	0.022	–0.014	0.024
8	–0.273****	0.023	0.019	0.024
9	–0.282****	0.022	–0.042	0.023
10	–0.501****	0.028	0.058	0.032
11	–0.288****	0.028	–0.016	0.030
12	–0.455****	0.033	0.003	0.037
13	–0.334****	0.020	–0.029	0.021
14	–0.444****	0.020	–0.153****	0.022
15	–0.357****	0.022	0.145****	0.023
16	–0.436****	0.027	–0.078**	0.030
17	–0.163****	0.033	–0.040	0.033
18	–0.413****	0.032	0.038	0.035
Genome-wide	–0.366****	0.005	0.003	0.006

****P < 0.0001; ***P < 0.001; **P < 0.01.

SE, Standard Error. The total number of SNPs was 29,315.

### Gene Content in Regions of High or Low Autozygosity or High *F_ST_*

After testing for autozygosity, the 2,000 SNPs with the highest LRT for *F_L_* were used to investigate their gene content using the Biomart software. This analysis revealed that loci with a high probability of autozygosity are clustered in regions that can expand up to 14 Mb in high autozygous regions (**Table 4**) and up to 11 Mb in regions of low autozygosity (**Table 5**). There were 1,093 and 907 SNPs in regions of high and low autozygosity, respectively. A Venn diagram that displays the numbers of common genes in regions for high and low autozygosity and with high differentiation is given in **Supplemental Figure 4**. The software PANTHER, using the function GO-complete biological process, was run separately for genes located in regions of high versus low autozygosity. Genes with a role in regulatory or vital metabolic functions were overrepresented in regions of high autozygosity (**Table 6**), while genes with a role in cell to cell signaling were overrepresented in regions of low autozygosity (**Table 7**). Genes involved in sensory perception were underrepresented in regions with both high and low autozygosity (**Tables 6** and **7**).

**Table 4:** Regions of high autozygosity (*F_L_*) in crossbreds based on the 2,000 SNPs with the highest LRT when testing the null hypothesis of equal autozygosity across each chromosome. Only regions with 10 or more SNPs are shown.

Chromosome	Number of SNPs	Position (bp)		Size (Mb)	*F_L_*
		Start	End		
1	19	69223005	71125466	1.90	0.37
1	58	230206948	244936885	14.7299	0.49
2	16	57528812	62015620	4.4868	0.37
2	21	64724523	73144365	8.4198	0.35
3	17	28373428	30457204	2.0838	0.22
4	13	62778825	64678175	1.8994	0.24
6	73	45460886	59527435	14.0665	0.39
6	52	71540166	75754270	4.2141	0.38
7	20	80190951	83511164	3.3202	0.47
7	65	108650887	112887383	4.2365	0.33
8	35	61039896	72304704	11.2648	0.34
8	15	148159458	148484852	0.3254	0.37
9	21	33065256	35935392	2.8701	0.25
10	19	35740932	38228609	2.4877	0.26
11	18	34724908	41674293	6.9494	0.43
12	38	286142	3770596	3.4845	0.44
13	25	101537295	107213278	5.676	0.36
13	23	161375471	166295270	4.9198	0.26
13	31	171295263	182416153	11.1209	0.31
14	29	46614469	51500676	4.8862	0.34
14	22	71358235	73600295	2.2421	0.36
14	23	77050539	78528923	1.4784	0.32
14	59	104302764	113333663	9.0309	0.45
15	11	9381966	10336687	0.9547	0.32
15	34	29248014	31207673	1.9597	0.43
15	33	90455132	93955844	3.5007	0.33
15	15	105914299	108268557	2.3543	0.33
15	40	111751664	116889115	5.1375	0.35
16	37	54241879	57813809	3.5719	0.28
18	77	38238900	45313098	7.0742	0.30

**Table 5:** Regions of low autozygosity (*F_L_*) in crossbreds based on the 2,000 SNP with the largest LRT when testing the null hypothesis of equal autozygosity across each chromosome.

Chromosome	Number of SNPs	Position		Size (Mb)	*F_L_*
		Start	End		
1	11	8348389	9118932	0.7705	0.0233
1	14	11633139	14253409	2.6203	0.0318
1	23	32278856	34960380	2.6815	0.0307
1	11	148618101	152957910	4.3398	0.0282
1	46	282649558	286524654	3.8751	0.0251
1	35	305220097	308379812	3.1597	0.0226
2	106	16416	11238061	11.2216	0.0189
2	75	15632627	20051590	4.419	0.0194
2	17	22473719	23411796	0.9381	0.0302
2	25	25506959	27954252	2.4473	0.0337
2	18	34594911	36317625	1.7227	0.0304
2	11	133135994	133626412	0.4904	0.0235
2	28	142568860	147165797	4.5969	0.0251
2	11	149538716	149861716	0.323	0.0325
6	11	35747679	37917319	2.1696	0.0227
6	21	143696009	147223902	3.5279	0.0124
6	16	151150412	157722783	6.5724	0.0208
7	75	4326388	7680759	3.3544	0.0076
7	25	120069590	121774453	1.7049	0.0059
7	24	126588087	128464615	1.8765	0.0039
8	38	18318655	19873562	1.5549	0.0132
8	30	140493989	144015324	3.5213	0.0140
14	49	167845	6321435	6.1536	0.0181
15	17	35765213	37678374	1.9132	0.0270
15	68	148187190	152769017	4.5818	0.0135
16	12	84977952	86024383	1.0464	0.0024

**Table 6:** GO complete biological processes that are significantly over- (OverR +) or under (OverR -) represented in regions of high autozygosity. Only regions with 10 or more SNPs were used.

Regions of High Autozygosity				
PANTHER GO Biological Process complete	Observed	Expected	OverR	*P* value
response to organic substance	124	76.62	+	8.52E-04
regulation of metabolic process	298	231.21	+	6.47E-03
regulation of biological quality	169	117.02	+	5.96E-03
cell surface receptor signaling pathway	106	67.33	+	2.48E-02
regulation of localization	127	83.59	+	1.50E-02
negative regulation of metabolic process	139	90.94	+	3.43E-03
regulation of response to stimulus	175	116.71	+	3.25E-04
negative regulation of cellular process	210	145.83	+	2.51E-04
positive regulation of cellular process	234	168.03	+	5.15E-04
negative regulation of biological process	228	160.42	+	1.59E-04
positive regulation of biological process	253	185.14	+	6.28E-04
regulation of nitrogen compound metabolic process	273	208.96	+	7.66E-03
negative regulation of cellular metabolic process	129	83.8	+	6.45E-03
regulation of cellular metabolic process	288	218.09	+	1.31E-03
negative regulation of macromolecule metabolic process	128	83.49	+	8.72E-03
metabolic process	445	369.22	+	7.56E-03
biological_process	894	818.52	+	6.11E-04
positive regulation of nucleobase-containing compound metabolic process	107	62.55	+	5.01E-04
system process	53	94.93	–	5.14E-03
detection of stimulus	11	61.66	–	4.73E-12
detection of chemical stimulus involved in sensory perception of smell	1	55.26	–	9.17E-20
detection of chemical stimulus involved in sensory perception	2	56.62	–	6.78E-19
detection of stimulus involved in sensory perception	5	58.2	–	5.50E-16
nervous system process	28	78.61	–	8.06E-08
detection of chemical stimulus	3	57.62	–	5.05E-18
sensory perception of smell	2	56.15	–	1.10E-18
sensory perception of chemical stimulus	3	58.88	–	1.43E-18
sensory perception	13	69.58	–	1.50E-13

**Table 7:** GO complete biological processes that are significantly over- (OverR +) or under (OverR -) represented in regions of low autozygosity. Only regions with 10 or more SNPs were used.

Regions of Low Autozygosity				
PANTHER GO Complete	Observed	Expected	OverR	*P* value
Biological Process complete				
cell-cell signaling	31	11.73	+	8.93E-03
positive regulation of biological process	119	73.13	+	1.67E-04
cell-cell adhesion via plasma-membrane adhesion molecules	15	3.01	+	3.72E-03
cell-cell adhesion	23	5.06	+	2.14E-05
response to radiation	19	4.85	+	4.60E-03
detection of stimulus	4	24.36	–	1.78E-03
detection of stimulus involved in sensory perception	3	22.99	–	9.49E-04

Analysis of the 2,000 SNPs with the highest *F_ST_* revealed chromosomal regions of high genetic differentiation between Landrace and Large White (**Supplemental Material Table 1**). These regions were overrepresented for genes involved in regulatory biological processes and also for genes involved in biological functions with productive interest, such as tissue development, anatomical structure, or animal organ development (**Supplemental Material Table 2**). These regions were underrepresented for sensory perception and neurological system processes, among others (**Supplemental Material Table 2**).

## Discussion

Swine breeds were developed after domestication by geographical and genetic isolation. Over time, genetic drift, natural, and artificial selection altered the genetic makeup of each swine breed. In this article, we propose the use of crossbreds and SNP arrays to identify genetic changes that occurred in two distinct breeds of swine. Two genetic analyses were performed: 1) analysis of ROHs and autozygosity in crossbreds, and 2) analysis of Δ and *F_ST_* as estimates of genetic differentiation. These two approaches, autozygosity and genetic differentiation, provide complementary information on the evolution of the parental breeds.

### Autozygosity in Landrace and Large White Crossbreds

An observed ROH in crossbreds indicates that the corresponding haplotype has persisted in the two breeds, in spite of opportunities to break it down by recombination. The number of generations back to the common ancestor was of about 46, 414, and 388 years when considering the distribution of ROHs with a minimum of 25, 15, and 5 SNPs, respectively. These results illustrate the large impact that the assumed number of contiguous and homozygous SNPs to declare a ROH has on estimating age to coalesce to a common ancestor. Historic records dated at the end of 19th century the creation of the parental breeds (Jones, 1998; Amills et al., 2010), which is consistent with ROH fragments with a minimum of 15 or 5 SNPs. Nevertheless, there were large ROHs that are not consistent with a fully genetic isolation between the parental breeds. It could be attributed to a combination of 1) larger realized generation interval before the 1950s, 2) not fully closed breeding within each of the parental breeds, and 3) a strong reduction in effective size at the time of the creation of the breeds followed by natural or artificial selection within breed for the same genotypes (Bosse et al., 2015). Selection may have favored the same allele at a gene within a chromosomal region in both breeds, and preserved the same haplotype in the two breeds by hitchhiking (Smith and Haigh, 1974). Thus, selection can lead to selective sweeps, which results in a reduction or elimination of variation among the nucleotides near a favorable mutation (Smith and Haigh, 1974; Kaplan et al., 1989). A widely used measure for estimating recent selection sweeps is extended haplotype homozygosity (EHH), defined as the probability that two randomly chosen chromosomes that carry the core haplotype (a collection of closely linked SNPs of interest) are identical by descent for the entire interval from the core region to a given point. EHH detects the transmission of an extended haplotype without recombination. A test for positive selection involves finding a core haplotype with a combination of high frequency and high EHH, compared with other core haplotypes. This method was extended in the so-called iHS statistic, which accounts for the allele frequencies in the core haplotype (Voight et al., 2006). Our results show that higher autozygosity is expected in some chromosomal regions than others. Therefore, this could have an effect on the detection of selection signatures with the EHH or iHS methods, which assume the same probability of autozygosity along each chromosome.

### Relationship Between *F_L_*, *F_ST_*, and Δ With Distal Chromosomal Distance

Our results show that the probability of autozygosity was very different between chromosomes and also along chromosome locations. This may be caused by chromosome structures that facilitate or obstruct recombination in different chromosomal regions. Our results identified positive correlations between the probability of autozygosity and distance to the closest distal part of the chromosome, although the size of this correlation varied greatly among chromosomes. This is consistent with previous work that observed larger recombination rates at the extremes than in the center of chromosomes in the pig (Muñoz et al., 2012). Chromosomal regions with a high recombination rate break down long haplotypes, which results in shorter identical-by-descent segments. In addition, centromeres are specialized regions that facilitate the binding of the kinetochore, which in turn attaches the chromosomes to microtubules during meiosis (Przewloka and Glover, 2009). Due to their essential function during meiotic segregation, the centromere and pericentric region have lower recombination rates (Nambiar and Smith, 2016), which is expected to result in higher autozygosity for the pericentric region, consistent with the results of this study. In the pig’s karyotype, SSC1 to SSC5 pairs are sub metacentric, pairs SSC6 and SSC7 are sub telocentric; pairs SSC8 to SSC12 are metacentric; and the remaining six pairs SSC13 to SSC18 are telocentric. Nevertheless, other studies show that the regions exhibiting higher levels of recombination tend to cluster around the ends of the chromosomes irrespective of the location of the centromere (Tortereau et al., 2012). On the contrary, the correlation of *F_ST_* and Δ with distal chromosomal distance was not consistent across chromosomes, suggesting that genetic drift and/or selection may have acted differently in the different chromosomes.

### Differentiation Between Landrace and Large White Breeds

The two measures of genetic differentiation that were used to investigate long-term changes in the genetic makeup of the two breeds at each SNP were: 1) estimates of the difference in allele frequency in the two breeds, and 2) the *F_ST_* statistic. Since both of these measures require estimates of allele frequencies, we developed a maximum likelihood approach to estimate allele frequencies and their sampling variance using genotype information from crossbreds. This method relies on departures from Hardy-Weinberg equilibrium conditions in crossbreds that occur when the allele frequencies at a SNP are different in the two parental breeds. Therefore, any factor that alters the allele frequencies among the gametes (when compared to the frequencies in the populations where they originated from) may affect estimation of allele frequencies with our method. This may include artificial and/or natural selection in the parental lines, the effects of which should be small unless selection is strong. The maximum likelihood method allows estimation of allele frequencies but is unable to distinguish which allele frequency corresponds to which parental population. Our interest was to investigate allele frequency differences between the parental breeds at each SNP position and neither Δ or *F_ST_* requires the assignment of allele frequencies to the parental breeds. Contrary to the *F_ST_* statistics, the difference in allele frequency between the two breeds, Δ, has the advantage that significance can be tested using a simple likelihood ratio test. This measure could also be used to test the dominance theory of heterosis. For example, SNPs with a high Δ could be tested for superiority of the heterozygotes in the parental breeds.

The *F_ST_* statistic has been widely used as a measure of genetic differentiation. This statistic has limitations for SNPs when the minor allele frequency is small. Jakobsson et al. examined the relationship between *F_ST_* and the frequency of the most frequent allele, demonstrating that the range of values that *F_ST_* can take is restricted considerably by the allele frequency distribution (Jakobsson et al., 2013). Genome-wide testing for *F_ST_* in the present study did not account for this dependence and, therefore, low values of this statistic may be due to a low minor allele frequency. Nevertheless, only 2.3% of the SNPs used in this study had a frequency of less than 0.05.

### Correlation Between Local Recombination Rate and *F_L_* and *F_ST_* Across the Swine Genome

The relationship between ROH density and recombination rate has been previously reported in pigs (Bosse et al., 2012). Our results strongly support a negative correlation between probability of recombination and probability of autozygosity even if maps of recombination rate and autozygosity were constructed with different animals with a different breed composition. It validates that autozygosity is not randomly distributed across the swine genome but that it is constrained by structural characteristics of chromosomes related to recombination hot- and cold-spots. A high recombination rate means that identical-by-descent segments will be shorter. The association between observed ROH density with recombination could be a result of one or both of the following mechanisms: 1) reduced effective population size arising from some form of linked selection (which extends farther across a chromosome in regions with low recombination rate) or 2) high power of detection of ROH for a given age to coalescence to a common ancestor in regions with lower recombination rate.

All together, these results may have implications in the applications of breeding in animal populations using molecular markers. The inbreeding coefficient of an individual is defined as the probability that two alleles at a locus in that individual are identical-by-descent (Crow and Kimura, 1970). At the molecular level, we showed that this probability depends on the location of each molecular marker, which is related to the local recombination rate. Therefore, these results may have implications in current methods to estimate molecular co-ancestry, inbreeding depression or breeding value estimation using genomic selection. All molecular approaches treat each locus equally.

The correlation between recombination rate and *F_ST_* did not show any general trend over chromosomes. The correlation was negative or positive, depending on the chromosome. This is likely due to selection history and genetic drift in the parental populations, which may have acted differently in the different chromosomes. However, the correlation between *F_L_* and *F_ST_* was negative with a low value, but significant as expected. The relationship between *F_L_* and *F_ST_* in each autosomal chromosome followed the same pattern since not high values of both parameters were observed in any chromosome. This could occur because of either genetic drift, purifying selection over very long time periods, or positive selection associated with the creation of the two parental breeds.

### Gene Content in Regions of High and Low Autozygosity and High *F_ST_*

Genes in chromosomal regions for either high or low *F_L_* or high *F_ST_* showed an overrepresentation or underrepresentation of genes related to some biological processes. Natural and/or artificial selection may have acted on the same haplotypes in those regions (high autozygosity) or on different haplotypes (high *F_ST_*) in the development of the two breeds. Our results show that high autozygosity in the crossbreds should correspond with low values of genetic differentiation (*F_ST_*) across the swine genome. In fact, the correlation between *F_ST_* and *F_L_* was negative and significant. Genes involved in biological functions, such as tissue development, anatomical structure, and animal organ development, were overrepresented in regions with high F*_ST_*. This can be attributed to long-term effects of selection for those traits, which may have been different for the two breeds. Genes involved in sensory perception, response to stimulus, and neurological system processes were underrepresented for significant regions based on all statistics (autozygosity and genetic differentiation) investigated. Previous work has shown that genes associated with immune response and olfaction exhibit fast evolution (Groenen et al., 2012). Nevertheless, all results regarding gene content presented here must be taken with caution since identification and location of genes in the current swine map may be incomplete or contain errors.

### Final Remark

We have illustrated that the genome of crossbreds contains genetic information (autozygosity and genetic differentiation) that may help to understand the evolution of the parental breeds. The application of this research to crosses between different species, such as the mule, may reveal the presence of ROH in the cross, which opens new avenues to investigate the life history of horse and donkey.

## Conclusions

Genetic analyses of crossbreds can reveal the location of common haplotypes (regions of autozygosity) and areas of genetic differentiation in the genomes of genetically distant breeds such as Landrace and Large White. Estimation of age of coalesce is influenced by the assumed minimum number of SNP in declared ROHs. Autozygosity is not uniform throughout the genome of crossbreds but more prominent in the central than in the distal part of chromosomes. Autozygosity is highly related to their chromosomal location which in turn depends on recombination hot and cold spots that can be tracked by the use of recombination maps. Genetic differentiation can be ascertained after estimation of allele frequencies in the parental lines, even when only crossbred genotype information is available. About 50% of the SNPs segregated at similar allele frequencies in the two parental breeds.

## Data Availability

The data that support the findings of this study are accessible from the PRRS Host Genetics Consortium, but restrictions apply to the availability of these data, which were used under license for the current study, and so are not publicly available. Data may be available from the authors upon reasonable request and with permission of the PRRS Host Genetics Consortium.

## Ethics Statement

All experimental protocols for these trials were approved by the Kansas State University Institutional Animal Care and Use Committee.

## Author Contributions

LG-R developed methods, carried out analysis, and wrote the first version of the manuscript. JDe made major contributions in the writing of the manuscript and was involved in the generation of the data used in this article. JDu helped in the implementation of the methods and in the editing of the data. WR contributed in the analyses of data and in the writing of the manuscript. All authors read and approved the final manuscript.

## Funding

Generation of these data was supported by the USDA ARS and NIFA award 2013-68004-20362. WMR and LGR acknowledge financial support from the Salvador de Madariaga program of the Ministry of Education of Spain, and AGL2016-75942-R and ERA-NET SUSPIG projects.

## Conflict of Interest Statement

The authors declare that the research was conducted in the absence of any commercial or financial relationships that could be construed as a potential conflict of interest.

## Supplementary Material

Supplemental material Figure 1 Lod Score plot of autozygosity of Landrace x Large White crossbreds.

Supplemental material Figure 2 Manhattan plot of Δ (difference in allele frequency) of Landrace and Large White. The dotted horizontal line represents the significance level at P < 0.001 after accounting for multiple testing using Bonferroni correction. The y-axis represents the LRT for each for the 18 swine chromosomes. The x-axis indicates the SNP position.

Supplemental material Figure 3 Map of the *F_ST_* statistic versus *F_L_* by chromosome. The y-axis represents *F_ST_* for each SNP within the 18 swine chromosomes. The x-axis is *F_L_* for the corresponding to the same SNP position.

Supplemental material Figure 4 Venn diagram for genes in regions with high *F_L_*, low *F_L_*, and high *F_ST_*.

Supplemental File 1 Autozygosity map including 32,533 SNPs. The columns are: SNP: name of the SNP; nchro: # of chromosome; *F_L_*: Probability of autozygosity of the SNP; 1 F_L(NULL)_: probability of autozygosity of the chromosome; position.v11: position in map version 11.1 Sscrofa; position.v10: position in map version 10 Sscrofa.

## Appendix

***Maximum likelihood estimation of allele frequencies in parental lines using genotype information from crossbreds***

A common situation in farm animals is that genotypic information is available from crossbreds but it is not available from the parental lines at crossing. Estimation of allele frequencies in the populations can be estimated by assuming that the parental populations are in Hardy-Weinberg equilibrium with allele frequencies f_A_ and f_B_. The expected frequency of heterozygotes in the crossbred population is $f_{A}\left(1-f_{B}\right)+f_{B}\left(1-f_{A}\right)$ and of the two homozygotes is f_A_f_B_ and (1 – f_A_) (1 – f_B_). The likelihood equation to estimate line allele frequencies is:

$$\begin{array}{l} L(f_{A},\;f_{B}|N_{11},N_{12},\;N_{12})=K\;{\left(f_{A}f_{B}\right)}^{N_{11}}\\ {(f_{A}(1-f_{B})\;+\;f_{B}(1-f_{A}))}^{N_{12}}{\left(\left(1-f_{A}\right)\left(1-f_{B}\right)\right)}^{N_{22}}, \end{array}$$

where *N_11_*, *N_21_*, and *N_22_* are the genotypic counts of the three genotypes in the crossbreds; and *K* is a constant.

After taking natural logarithm the above equation yields

$$\begin{array}{l} ln\;L(f_{A},\;f_{B}\text{|}N_{11},N_{12},\;N_{22}\text{)}=N_{11}\;ln\;\left(f_{A}f_{B}\right)\\ +\;N_{12}ln{\left(f_{A}\left(1-f_{B}\right)+f_{B}\left(1-f_{A}\right)\right)}^{}+\;\;N_{22}ln{\left(\left(1-f_{A}\right)\left(1-f_{B}\right)\right)}^{} \end{array}$$

Maximizing the above equation was carried out using the grid search method. There were always two maxima corresponding to estimates of the allele frequencies coming from either population at crossing. That is, we can estimate allele frequencies in the parental populations but we cannot know which of the two corresponds to each breed. Hypothesis testing for the difference in allele frequencies between the parental populations was carried out using a likelihood ratio test:

$$\begin{array}{l} LRT=2[ln\;L\;(f_{A}^{Max},\;f_{B}^{Max}\text{|}N_{11},N_{12},\;N_{22}\text{)}\\ -\;ln\;L\;(f_{A}^{o},\;f_{B}^{o}\text{|}N_{11},N_{12},\;N_{22})], \end{array}$$

where $f_{A}^{Max}$ and $f_{B}^{Max}$ are the allele frequencies estimates in populations A and B, respectively; $f_{A}^{o}$ and $f_{B}^{o}$ are the allele frequencies under the null hypothesis of equal frequencies in populations A and B, respectively.

Sampling variances of the estimates of the allele frequencies was performed by taking the first and second derivatives of the likelihood function with respect to allele frequencies:

$$\begin{array}{c} \frac{\;\partial ln\;L(f_{A},\;f_{B}\text{|}N_{11},N_{12},\;N_{22}\text{)}}{\partial \;f_{A}}=\frac{N_{11}}{f_{A}}-\frac{N_{22}}{\left(1-f_{A}\right)}+\frac{N_{12}\left(1-2f_{B}\right)}{f_{A}+f_{B}-2f_{A}f_{B}\;}\\ \frac{\;\partial ln\;L(f_{A},\;f_{B}\text{|}N_{11},N_{12},\;N_{22}\text{)}}{\partial \;f_{B}}=\frac{N_{11}}{f_{B}}-\frac{N_{22}}{\left(1-f_{B}\right)}+\frac{N_{12}\left(1-2f_{A}\right)}{f_{A}+f_{B}-2f_{A}f_{B}}\\ \frac{\;\partial^{2}ln\;L(f_{A},\;f_{B}\text{|}N_{11},N_{12},\;N_{22}\text{)}}{\partial^{2}f_{A}}=-\frac{N_{11}}{f_{A}^{2}}-\frac{N_{22}}{{\left(1-f_{A}\right)}_{}^{2}}-\frac{N_{12}{\left(1-2f_{B}\right)}^{2}}{{\left[f_{A}+f_{B}-2f_{A}f_{B}\right]}^{2}}\\ \frac{\;\partial^{2}ln\;L(f_{A},\;f_{B}\text{|}N_{11},N_{12},\;N_{22}\text{)}}{\partial^{2}f_{B}}=-\frac{N_{11}}{f_{B}^{2}}-\frac{N_{22}}{{\left(1-f_{B}\right)}_{}^{2}}-\frac{N_{12}{\left(1-2f_{A}\right)}^{2}}{{\left[f_{A}+f_{B}-2f_{A}f_{B}\right]}^{2}}\\ \frac{\;\partial^{2}ln\;L(f_{A},\;f_{B}\text{|}N_{11},N_{12},\;N_{22}\text{)}}{\partial f_{A}\partial f_{B}}=-\frac{N_{12}\left[2\left(f_{A}+f_{B}-2f_{A}f_{B}\right)+\left(1-2f_{A}\right)\left(1-2f_{B}\right)\right]}{{\left[f_{A}+f_{B}-2f_{A}f_{B}\right]}^{2}}\\ \frac{\;\partial^{2}ln\;L(f_{A},f_{B}\text{|}N_{11},N_{12},\;N_{22}\text{)}}{\partial f_{B}\partial f_{A}}=-\frac{N_{12}\left[2\left(f_{A}+f_{B}-2f_{A}f_{B}\right)+\left(1-2f_{A}\right)\left(1-2f_{B}\right)\right]}{{\left[f_{A}+f_{B}-2f_{A}f_{B}\right]}^{2}} \end{array}$$

Therefore, the Hessian matrix is

$$H\left(f_{A},f_{B}\right)=\left[\begin{array}{ll} \frac{\;\partial^{2}ln\;L(f_{A},\;f_{B}\text{|}N_{11},N_{12},\;N_{22}\text{)}}{\partial^{2}f_{A}} & \frac{\;\partial^{2}ln\;L(f_{A},\;f_{B}\text{|}N_{11},N_{12},\;N_{22}\text{)}}{\partial f_{A}\partial f_{B}}\\ \frac{\;\partial^{2}ln\;L(f_{A},\;f_{B}\text{|}N_{11},N_{12},\;N_{22}\text{)}}{\;\partial f_{B}\partial f_{A}} & \frac{\;\partial^{2}ln\;L(f_{A},\;f_{B}\text{|}N_{11},N_{12},\;N_{22}\text{)}}{\partial^{2}f_{B}} \end{array}\right]$$

The matrix of the sampling variance of the estimates of allele frequencies is

$$V\left(f_{A},f_{B}\right)=[-E{\left(H\left(f_{A},f_{B})\right)\right]}^{-1}$$

The standard errors are just the square roots of the diagonal terms in this variance-covariance matrix.
