# Supplementary figures and images for: Autozygosity and Genetic Differentiation of Landrace and Large White Pigs as Revealed by the Genetic Analyses of Crossbreds
Source: Front Genet. 2019 Sep 5;10:739. doi: 10.3389/fgene.2019.00739 (PMC6739446; doi:10.3389/fgene.2019.00739)

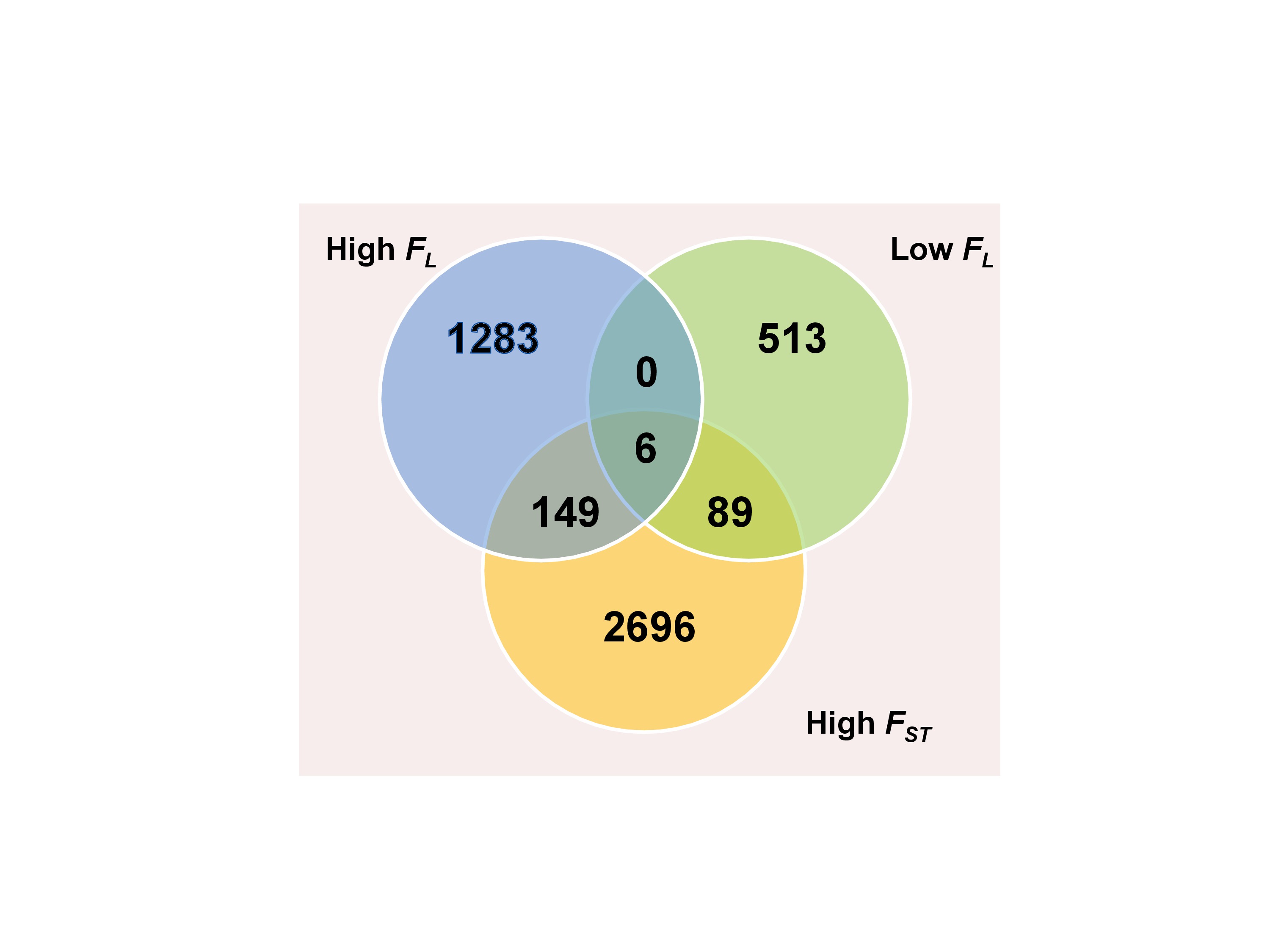

Supplement: Supplemental material Figure 4 — Venn diagram for genes in regions with high FL, low FL, and high FST. [file Image_4.jpeg]
