# Supplementary material for: Autozygosity and Genetic Differentiation of Landrace and Large White Pigs as Revealed by the Genetic Analyses of Crossbreds
Source: Front Genet. 2019 Sep 5;10:739. doi: 10.3389/fgene.2019.00739 (PMC6739446; doi:10.3389/fgene.2019.00739)
Supplement: Supplementary file 6 [file Table_1.docx]

**Supplemental Material Table 1. Chromosomal regions for the 2000 SNPs with highest *F_ST_***_._ Only regions with 10 or more markers are shown.

| Chromosome | #markers | Start | End | Size (Mb) | Average *F_ST_* |
| --- | --- | --- | --- | --- | --- |
| 1 | 53 | 493510 | 22292508 | 21.8 | 0.22 |
| 1 | 15 | 25275233 | 34468175 | 9.19 | 0.21 |
| 1 | 15 | 233565583 | 240843268 | 7.28 | 0.21 |
| 1 | 17 | 248440959 | 257077784 | 8.64 | 0.21 |
| 1 | 27 | 264235138 | 268985154 | 4.75 | 0.22 |
| 2 | 13 | 13620582 | 21629661 | 8.01 | 0.21 |
| 2 | 21 | 123270341 | 132424131 | 9.15 | 0.21 |
| 2 | 17 | 134743244 | 142401364 | 7.66 | 0.22 |
| 3 | 21 | 8241847 | 15805383 | 7.56 | 0.21 |
| 3 | 20 | 20437306 | 32256023 | 11.82 | 0.23 |
| 3 | 19 | 45069392 | 56175124 | 11.11 | 0.19 |
| 3 | 11 | 117415922 | 120659876 | 3.24 | 0.19 |
| 4 | 11 | 11878242 | 17231747 | 5.35 | 0.21 |
| 4 | 28 | 81229506 | 89283931 | 8.05 | 0.22 |
| 4 | 42 | 96175348 | 109875916 | 13.7 | 0.26 |
| 5 | 17 | 61559633 | 67662549 | 6.1 | 0.20 |
| 5 | 15 | 70146755 | 76859809 | 6.71 | 0.22 |
| 5 | 12 | 92864624 | 99052363 | 6.19 | 0.24 |
| 6 | 12 | 16539345 | 17729820 | 1.19 | 0.18 |
| 6 | 25 | 25753694 | 32407613 | 6.65 | 0.22 |
| 6 | 16 | 155269108 | 163647877 | 8.38 | 0.21 |
| 7 | 15 | 24986445 | 30265671 | 5.28 | 0.18 |
| 7 | 18 | 54698134 | 63522661 | 8.82 | 0.22 |
| 7 | 18 | 93130604 | 101464925 | 8.33 | 0.24 |
| 7 | 12 | 108575927 | 111283606 | 2.71 | 0.19 |
| 7 | 14 | 113296814 | 115653097 | 2.36 | 0.20 |
| 8 | 19 | 2375393 | 9557080 | 7.18 | 0.21 |
| 8 | 12 | 15986265 | 21808575 | 5.82 | 0.22 |
| 8 | 21 | 27902238 | 39471996 | 11.57 | 0.23 |
| 9 | 22 | 5717797 | 18855958 | 13.14 | 0.20 |
| 9 | 17 | 49752735 | 56474976 | 6.72 | 0.23 |
| 9 | 35 | 124337182 | 139401727 | 15.06 | 0.21 |
| 10 | 17 | 19909598 | 28911984 | 9 | 0.21 |
| 10 | 16 | 37747137 | 44833617 | 7.09 | 0.24 |
| 10 | 34 | 47530718 | 62344578 | 14.81 | 0.21 |
| 11 | 26 | 818680 | 15309309 | 14.49 | 0.22 |
| 11 | 27 | 45895693 | 52354280 | 6.46 | 0.23 |
| 11 | 23 | 54445816 | 63272581 | 8.83 | 0.24 |
| 13 | 11 | 3338185 | 8805014 | 5.47 | 0.24 |
| 13 | 20 | 81193216 | 85566377 | 4.37 | 0.20 |
| 13 | 12 | 106648129 | 115450857 | 8.8 | 0.23 |
| 13 | 11 | 132254648 | 138098546 | 5.84 | 0.20 |
| 13 | 41 | 191137012 | 208240759 | 17.1 | 0.19 |
| 14 | 15 | 54635688 | 59378486 | 4.74 | 0.19 |
| 14 | 61 | 63386683 | 74094388 | 10.71 | 0.22 |
| 14 | 41 | 115969255 | 132845711 | 16.88 | 0.18 |
| 15 | 12 | 1151527 | 6334141 | 5.18 | 0.22 |
| 15 | 27 | 121133797 | 130568643 | 9.43 | 0.20 |
| 15 | 15 | 134748950 | 140089805 | 5.34 | 0.19 |
| 16 | 11 | 1493104 | 5509256 | 4.02 | 0.22 |
| 16 | 25 | 31240810 | 38067633 | 6.83 | 0.19 |
| 16 | 15 | 52806719 | 56799073 | 3.99 | 0.16 |
| 17 | 25 | 19746778 | 30437824 | 10.69 | 0.22 |
| 17 | 26 | 51898192 | 61462919 | 9.56 | 0.23 |
