# Supplementary material for: Autozygosity and Genetic Differentiation of Landrace and Large White Pigs as Revealed by the Genetic Analyses of Crossbreds
Source: Front Genet. 2019 Sep 5;10:739. doi: 10.3389/fgene.2019.00739 (PMC6739446; doi:10.3389/fgene.2019.00739)
Supplement: Supplementary file 7 [file Table_2.docx]

**Supplemental Material Table 2. GO complete biological processes that are significantly over- (OverR +) or under- (OverR -) represented in regions of high *F_ST_*.**

| **Regions of High F_ST_** |  |  |  |  |
| --- | --- | --- | --- | --- |
| PANTHER GO-Complete Biological Process | Observed | Expected | OverR | P value |
| developmental process | 506 | 351.83 | + | 1.02E-13 |
| positive regulation of biological process | 545 | 390.95 | + | 1.40E-12 |
| positive regulation of cellular process | 499 | 354.83 | + | 9.64E-12 |
| anatomical structure development | 470 | 330.12 | + | 1.17E-11 |
| biological_process | 1893 | 1728.48 | + | 1.30E-11 |
| regulation of metabolic process | 645 | 488.25 | + | 5.36E-11 |
| negative regulation of biological process | 476 | 338.76 | + | 6.33E-11 |
| regulation of macromolecule metabolic process | 604 | 454.34 | + | 1.88E-10 |
| multicellular organism development | 424 | 297.87 | + | 4.43E-10 |
| regulation of cellular metabolic process | 607 | 460.54 | + | 7.82E-10 |
| regulation of primary metabolic process | 593 | 449.46 | + | 1.45E-09 |
| negative regulation of cellular process | 431 | 307.95 | + | 3.08E-09 |
| regulation of nitrogen compound metabolic process | 581 | 441.26 | + | 4.09E-09 |
| system development | 383 | 268.84 | + | 8.86E-09 |
| cellular developmental process | 337 | 230.16 | + | 1.07E-08 |
| regulation of response to stimulus | 354 | 246.45 | + | 2.83E-08 |
| negative regulation of macromolecule metabolic process | 270 | 176.31 | + | 2.89E-08 |
| negative regulation of metabolic process | 289 | 192.04 | + | 3.06E-08 |
| cell differentiation | 325 | 223.96 | + | 8.04E-08 |
| biological regulation | 1220 | 1059.38 | + | 1.28E-07 |
| cellular process | 1474 | 1317.25 | + | 2.31E-07 |
| positive regulation of metabolic process | 342 | 240.8 | + | 2.62E-07 |
| positive regulation of cellular metabolic process | 325 | 227.39 | + | 4.41E-07 |
| negative regulation of cellular metabolic process | 263 | 176.97 | + | 1.07E-06 |
| animal organ development | 274 | 187.39 | + | 2.04E-06 |
| negative regulation of nitrogen compound metabolic process | 249 | 166.89 | + | 2.43E-06 |
| regulation of signaling | 310 | 218.53 | + | 2.86E-06 |
| positive regulation of macromolecule metabolic process | 315 | 223.29 | + | 3.60E-06 |
| positive regulation of nitrogen compound metabolic process | 309 | 218.53 | + | 4.27E-06 |
| regulation of cell communication | 306 | 216.31 | + | 5.02E-06 |
| regulation of multicellular organismal process | 259 | 177.41 | + | 7.67E-06 |
| tissue development | 165 | 101.39 | + | 1.08E-05 |
| anatomical structure morphogenesis | 215 | 143.28 | + | 2.76E-05 |
| localization | 517 | 407.91 | + | 3.15E-05 |
| negative regulation of gene expression | 173 | 110.15 | + | 5.02E-05 |
| regulation of signal transduction | 277 | 197.03 | + | 6.29E-05 |
| regulation of biological process | 1129 | 994.11 | + | 7.93E-05 |
| detection of chemical stimulus involved in sensory perception | 9 | 119.57 | - | 7.60E-37 |
| detection of chemical stimulus involved in sensory perception of smell | 9 | 116.69 | - | 1.25E-35 |
| sensory perception of smell | 12 | 118.57 | - | 3.00E-33 |
| detection of chemical stimulus | 14 | 121.67 | - | 1.44E-32 |
| detection of stimulus involved in sensory perception | 15 | 122.89 | - | 3.98E-32 |
| sensory perception of chemical stimulus | 16 | 124.33 | - | 8.60E-32 |
| detection of stimulus | 26 | 130.21 | - | 4.81E-26 |
| sensory perception | 38 | 146.94 | - | 3.98E-24 |
| G-protein coupled receptor signaling pathway | 76 | 182.51 | - | 1.71E-16 |
| nervous system process | 66 | 166 | - | 5.47E-16 |
| system process | 113 | 200.46 | - | 1.29E-08 |
